# Case of Poisoning by Arsenic, Successfully Treated

**Published:** 1821-11

**Authors:** Jos. Hume


### *Case of Poisoning by Arsenic, successfully treated.* By Jos. Hume, Esq.

THE following instance of speedy recovery from the effects of arsenic will, I trust, fully exemplify the favourable opinion I have already given of the probable advantages to be derived from the use of *carbonate of magnesia*, and my predilection for this earth as an antidote. This opinion, together with many valuable remarks by the author, connected with the detection of arsenic, will be found in Mr. Marshall's "Remarks on Arsenic," published about four years ago.

It was with reluctance I undertook the charge of this patient: I had, indeed, refused the application, recommending that it should be made to a professional gentleman whom I named; for, although originally destined, and in a great degree prepared, for the practice of medicine and surgery, I have long confined my attention principally to practical and scientific chemistry and pharmacy. After this apology, I now proceed with a very brief detail of the case; my avocations at present preventing my stating all that might be said upon the subject.

On the 18th of July last, M. N., a young married woman, about twenty years of age, in consequence of some violent altercation with her husband, a fruiterer, and she being then in a state of inebriety, immediately set about the means of self-destruction, by purchasing some arsenic at a neighbouring druggist's shop, pretending that it was for cleaning brass.

By subsequent information, I found that the arsenic was taken after supper, and at a late hour, being near midnight; that she had eaten rather heartily of cold fried sheep's liver; had taken neither bread nor any liquid with that meal; and that she swallowed the arsenic very soon after, having mixed it in a wine-glass with a little water, and "stirring it well up with a tea-spoon," as she informed me.

About eight o'clock on the following morning, the mother of this poor woman came to my house, and, in the most urgent and impressive manner, implored me to accompany her instantly to see her dying daughter, who had taken poison; at the same time presenting me with the paper containing the remainder of the arsenic. This parcel I, of course, retained in my possession, for the purpose of examination and analysis, in case an authorized inquiry by a jury should be necessary. As the distance was short, and as the wretched mother seemed most unwilling to lose time, as she expressed it, by sending for other assistance, I immediately attended her to her daughter's lodging; but with no expectation of success, as, without any nice examination of the powder, I soon discovered its nature, and the mother had informed me of the quantity which her daughter had taken.

On entering the room, I found the miserable victim suffering under the most excruciating agonies, attended with constant efforts to vomit or retchings, although it appeared that she had, in the early part of the morning, vomited once or twice rather copiously. The pulse was extremely rapid, and rather unequal; it was, however, above 120. In the commencement of such accidents, the pulse is, probably, not to be relied on as an indication; for, particularly on this occasion, the patient suffered much from mental as well as bodily agitation; seemed fully sensible of her danger, and, I am confident, repented most sincerely of the rashness of her conduct. She complained of the well-known signs attending an over-dose of arsenic; such as acute and fixed pain in and about the gastric region; a sensation of burning heat in the throat and fauces; and, in short, to use a convenient expression, she was labouring under all the symptoms peculiar to the arsenical virus.

I quickly returned home, requesting the mother to accompany me for the sake of dispatch, and prescribed the following mixture:—

℞. Magnesia carbonatis, ℥j.

Aquæ distillatæ, ℥xv.

Vini opii, ʒjss.

Spiritus lavand. comp. ʒiij.

Sacchari albi, ℥ss. M.

Capiat ægra cochlearia duo magna frequenter, phialâ assiduè agitatâ.

Besides a written label on the bottle, I instructed the mother to give a dose of the mixture every ten minutes, while the symptoms continued violent; but, should there be any material remission, then to exhibit the medicine every quarter of an hour or twenty minutes, according to circumstances.

So slight were my expectations of saving this unfortunate woman, that I did not think it requisite to visit her again. I had, indeed, informed the mother, as well as the unhappy husband, who blamed himself as having been the cause of his wife's situation, that I considered it a hopeless case. My attention now was rather occupied in preparing such evidence as might be satisfactory to a coroner's jury, than forming any plan for the treatment of the patient; but another visit from the mother checked this career. She informed me that her daughter "had *again* been taken very ill," and begged I would see her immediately.

This message convinced me there had been a remission of the symptoms, and that the same remedy ought to be continued; I therefore, not having it in my power at that time to visit the patient, desired that the daughter should persevere with the same medicine; observing to her, that I could prescribe nothing more efficacious. This was assented to, and the same mixture was repeated, omitting half a drachm of the vinum opii, the patient having been disposed to drowsiness by the first proportion.

I am unwilling to delay what I consider to be the chief object of my letter,—the mode of applying *magnesia*; but, having other circumstances to add, which may be important and, I hope, acceptable to the readers of your valuable Journal, and my time being at present too much engaged in professional pursuits to permit me to proceed any farther on this subject,—I shall only say, that the patient herself waited on me, I believe, on the fifth day after, and that, in less than ten days, she was perfectly recovered. It is proper to mention, that oleum ricini and other (what I shall call) *occasional* remedies, were also employed in this case.

*Long-Acre; Oct. 11th*, 1821.

---

